# Immunological Features of Respiratory Syncytial Virus-Caused Pneumonia—Implications for Vaccine Design

**DOI:** 10.3390/ijms18030556

**Published:** 2017-03-04

**Authors:** Emma Rey-Jurado, Alexis M. Kalergis

**Affiliations:** 1 Millennium Institute on Immunology and Immunotherapy, Departamento de Genética Molecular y Microbiología, Facultad de Ciencias Biológicas, Pontificia Universidad Católica de Chile, Santiago 8330644, Chile; erey@bio.puc.cl; 2 Departamento de Endocrinología, Facultad de Medicina, Pontificia Universidad Católica de Chile, Santiago 8330644, Chile

**Keywords:** human respiratory syncytial virus, pneumonia, host immunity, vaccines and therapies

## Abstract

The human respiratory syncytial virus (hRSV) is the causative agent for high rates of hospitalizations due to viral bronchiolitis and pneumonia worldwide. Such a disease is characterized by an infection of epithelial cells of the distal airways that leads to inflammation and subsequently to respiratory failure. Upon infection, different pattern recognition receptors recognize the virus and trigger the innate immune response against the hRSV. Further, T cell immunity plays an important role for virus clearance. Based on animal studies, it is thought that the host immune response to hRSV is based on a biased T helper (Th)-2 and Th17 T cell responses with the recruitment of T cells, neutrophils and eosinophils to the lung, causing inflammation and tissue damage. In contrast, human immunity against RSV has been shown to be more complex with no definitive T cell polarization profile. Nowadays, only a humanized monoclonal antibody, known as palivizumab, is available to protect against hRSV infection in high-risk infants. However, such treatment involves several injections at a significantly high cost. For these reasons, intense research has been focused on finding novel vaccines or therapies to prevent hRSV infection in the population. Here, we comprehensively review the recent literature relative to the immunological features during hRSV infection, as well as the new insights into preventing the disease caused by this virus.

## 1. Introduction

Pneumonia is defined as pathological inflammation of the lungs. According to the World Health Organization (WHO), pneumonia caused 920,136 child deaths in 2015, thereby making it a major public health burden worldwide [1]. Further, not only children are affected by pneumonia but also the elderly, with 53,000 deaths reported annually in the >65 years old population in the United States alone [2]. Clinical studies of childhood pneumonia contributed to the definition of pneumonia as a respiratory rate higher than 50/min, fever and chest in-drawing [3]. Symptoms of pneumonia may include chest pain, wheezing and breathing difficulties [4]. Pneumonia is primarily due to infectious agents, such as viruses, bacteria and fungi. *Streptococcus pneumoniae* and *Klebsiella pneumoniae* are the most common bacterial etiology, whereas the human respiratory syncytial virus (hRSV), influenza and rhinovirus are the more frequent viral causal agents of pneumonia [5,6]. Furthermore, virus and bacteria co-infections are usually observed in about 40% of patients with community-acquired pneumonia [7].

hRSV is one of the predominant causal viruses of pneumonia [8] and is also the main agent causing acute lower respiratory tract infections (ALRTIs), affecting children younger than five years old and the elderly [9]. Mild manifestations of hRSV include rhinorrhea, cough, congestion, low-grade fever, reduced appetite and respiratory distress [10]. However, hRSV can also cause severe symptoms, such as alveolitis, bronchiolitis and pneumonia [10]. Moreover, recently, extra pulmonary manifestations, such as encephalitis, cardiopathy and hepatitis have been reported [11,12,13,14]. Importantly, global mortality due to hRSV-associated ALRTI in children younger of 5 years is estimated to be from 66,000 to 199,000 deaths per year [15]. The cost in medical health expenses on hospitalizations due to hRSV infections has been estimated to be 394 million USD per year [16]. To note, risk factors associated to severe cases of hRSV include elderly, age under 2 or 3 months, premature birth, chronic lung disease, congenital heart disease, simultaneous infections [17,18] and immunosuppression [19,20]. Around 36% of individuals can be reinfected at least once during the winter season [21]. Along these lines, such reinfections could be caused by deficient humoral and cellular immunity response after the first virus encounter [21,22].

hRSV is an enveloped, negative sense, single stranded RNA virus belonging to the recently defined Pneumoviridae family, Orthopneumovirus genus. The viral genome is non-segmented RNA, 15.2 kb in length, which encodes eleven proteins and the following ten genes: *NS1-NS2-N-P-M-SH-F-G-M2-L* (from 3′ to 5′) [23,24]. The Fusion (F), Glycoprotein (G) and Small Hydrophobic (SH) proteins are expressed at the surface whereas the Nucleoprotein (N), Phosphoprotein (P), large polymerase protein (L), Matrix protein (M) and M2-1 proteins are below the virus envelope [25,26]. The F protein is in charge of the fusion of the viral envelope with the host membrane [27,28,29,30]. On the other hand, the G protein has been shown to mediate the attachment of hRSV to the cell membrane [31] and the fusion of the F protein with the host cell membrane [32]. In contrast, the SH protein has been defined as a viroporin, thereafter allowing the entrance of low molecular weight molecules and modifying the cell permeability [26,33]. The L, P, M2-1 and N hRSV proteins constitute the ribonucleoprotein (RNP), which encapsides the viral RNA [34,35]. From this complex, the L protein regulates the replication and transcription of the hRSV RNA and the N protein is thought to protect viral RNA from nucleases [23,36]. The P protein is essential for the RNP complex and its phosphorylation may be required for the virus replication in vitro and in vivo [34]. M2-1 protein, which also is part of the RNP complex, promotes the transcription of all hRSV genes [37]. Likewise, M2-2 protein mediates the “switch” from transcription to RNA replication [38]. Besides from structural proteins, the hRSV genome also includes two non-structural proteins called NS1 and NS2. Finally, as another hRSV protein, the matrix protein M that promotes viral assembly is required for hRSV replication.

Animal studies have shown that hRSV infection is characterized by the recruitment of infiltrating immune cells to the lungs, thereby producing lung damage and pulmonary inflammatory hyper reactivity. Such pulmonary inflammation is due to a Th-2 biased immune response, constituted of high levels of IL-4 and IL-13 cytokines. On the contrary, T helper (Th)-1 response, producing cytokines such as Interferon (IFN)-γ and IL-2, has been associated with disease control and virus clearance. While such cytokine patterns have been shown in animal models, infant patients from 3 weeks to 24 months of age with acute phase hRSV bronchiolitis showed a decrease of IFN-γ+ and an increase of IL-4+ CD4^+^ and CD8^+^ T cells [39]. Further, patients with hRSV bronchiolitis showed a decrease in the proportion of γδ T cells producing IFN-γ in response to mitogen stimulation [40].

Here, we discuss the host immune response associated to hRSV infection focusing on hRSV-associated pneumonia, the evasion of the immune response by the virus, as well as the prophylaxis and vaccines under development to prevent the disease caused by this pathogen.

## 2. Host Immune Response Associated to hRSV-Pneumonia

**Figure 1:**
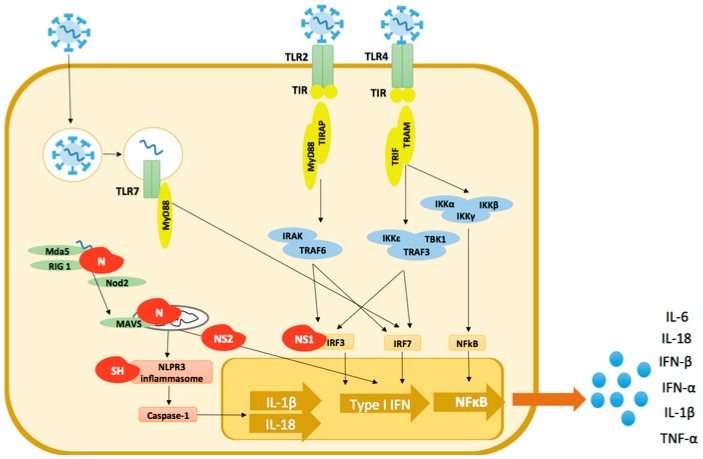
Mechanisms used by hRSV to evade host innate immunity. TLR, Toll-like receptor; RIG-I, retinoic acid-inducible gene-1; NOD, nucleotide-binding oligomerization domain; MDA5, melanoma differentiation-association gene 5; MAVS, mitochondrial antiviral signalling; NLPRP3, NOD-like receptor family pyrin domain containing 3; MyD88, Myeloid differentiation primary response gene 88; TIR, Toll-interleukin 1 receptor; TRAF, TNF receptor associated factor; IKK, Interleukin-1 receptor-associated kinase; IKKβ, inhibitor of nuclear factor κB kinase subunit beta; IRF, Interferon regulatory factor. The human respiratory syncytial virus (hRSV) is recognized by different pattern recognition receptors (PRRs) including TLR-2, TLR-4, TLR-7, RIG-I and NΟD2. hRSV can also enter via endocytosis and viral RNA can be recognized inside the endosome by TLR-7. Thereafter, a cascade of inflammatory response is activated. Different proteins of the hRSV have been found to interfere in the innate response against the infection. The nucleoprotein (N) has been suggested to “sequester” MDA5, RIG-I and MAVS proteins interfering in the type I IFN production. Besides from N, both non-structural (NS)1 and NS2 proteins have been shown to inhibit the IFN production at the MAVS and IRF3 level. On the other hand, the small hydrophobic (SH) protein has been shown to activate the NLPRP3 inflammasome, eventually triggering the production of IL-1β and IL-18.

hRSV is the major infectious agent for bronchiolitis, which is characterized by infection and inflammation of the distal bronchiolar airways. In addition, hRSV is also a causal agent of severe pneumonia. hRSV-caused pneumonia is characterized by the infection of alveolar lung regions, thereby triggering an alveolar inflammation that can eventually result in severe pulmonary disease with hypoxia and respiratory failure [41]. At the lungs, airway epithelial cells (AECs), macrophages and dendritic cells (DCs) represent the first cells encountering hRSV. Upon infection, different pattern recognition receptors (PRRs) in these cells trigger a downstream innate immune cascade, including Toll-like receptors (TLRs), retinoic acid-inducible gene I (RIG-I)-like receptor (RLR) family members and NOD-like receptors (NLR) [42] (Figure 1). At this point, different proteins of the hRSV have been found to interact with the host to modulate the innate immune response. The F protein has been shown to interact with several host proteins, such as Toll-like receptor (TLR) 4, intercellular adhesion molecule 1 (ICAM-1) and nucleolin [43,44]. Moreover, the SH protein has also been found to activate the NOD-like receptor family, pyrin domain containing 3 (NLRP3), thereby triggering the activation of the inflammasome [45]. On the other hand, the N protein was shown to colocalize and interact with RIG-I-like receptors, the mitochondrial antiviral signalling (MAVS) protein and the melanoma differentiation-associated gene 5 (MDA5), thereby modulating the downstream innate immune response [46] (Figure 1). Indeed, such study also demonstrated an interaction between MDA5 and N protein within inclusion bodies, suggesting that hRSV may be “sequestering” these important proteins required to trigger the triggering innate immune response [46]. As a result of MDA5 sequestration, a decrease of type I IFN production is observed, which hampers the control of hRSV infection. The NLRP3 receptor, a member of the NLR family, together with proteins, such as caspase-1 and the Pyrin domain containing NLRs known as NALP proteins, constitute the inflammasome responsible for the production of IL-1β and IL-18 [47,48]. Thus, it has been shown that hRSV triggers NLRP3 inflammasome by the SH protein [45] (Figure 1).

**Figure 2:**
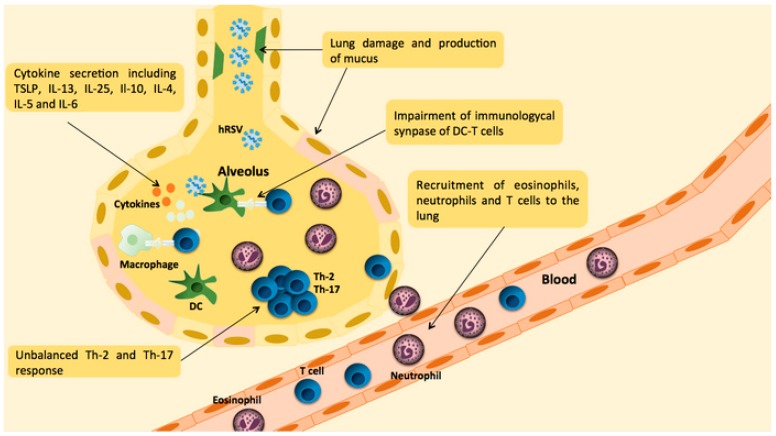
Airway inflammation due to hRSV infection. Upon hRSV infection, airway epithelial cells, dendritic cells and macrophages recognize hRSV. Such recognition triggers a downstream innate response, which eventually will result in the production of cytokines and chemokines such as thymic stromal lymphopoetin (TSLP), IL-13, IL-25, Il-10, IL-4, IL-5 and IL-6. Likewise, such hRSV infection promotes the recruitment of neutrophils, eosinophils and T cells. Such inflammatory response results in Th-2 and Th-7 response, mucus production and lung damage.

Such an hRSV infection promotes the recruitment of immune cells to the site of the challenge. Further, hRSV infection generates the virus-associated immunopathology characterized by up-regulation of proinflammatory cytokines including thymic stromal lymphopoetin (TSLP), Interleukin (IL)-4, IL-6, IL-10 and IL-13 and infiltration of mononuclear cells (mainly T cells), neutrophils and eosinophils (Figure 2) [49,50,51,52]. These cytokines fail to achieve hRSV clearance, herein favoring the persistence of the virus. To note, the TSLP together with the epithelial cell-derived IL-7, an IL-7-like cytokine, IL-25 and IL-33 are characteristic of acute asthma exacerbations and Th-2 polarized response during virus infections [53]. Likewise, increased expression of TSLP has been found in asthmatic children after hRSV infection [54]. Consistent with this notion, studies in animal models suggest that hRSV infection could predispose to asthma, however additional research would be needed to clearly understand such an association [55]. Further, TSLP has been shown in mice to induce functional maturation of myeloid DCs (mDCs) [50] and an increased expression of molecules that polarize to a Th-2 response. It is also thought that hRSV can directly impair the function of chemokines and cytokines because the G protein colocalizes with host proteins, such as CX3CR1 in ciliated lung cells [56]. Such pathogenic inflammation leads to an unbalanced Th-2 and Th-17 response with low production of interferon (IFN)-γ, which contributes to lung damage and ameliorates virus clearance [57,58]. Most of these data are derived from animal models, however low production of IFN-γ has also been observed in patients suffering from hRSV bronchiolitis [39,40]. Importantly, the IL-17A produced at the airways after hRSV infection has been linked to neutrophil infiltration to the lungs [59]. Likewise, high levels of IL-17 have been related with hRSV bronchiolitis [60] and with community-acquired pneumonia [61].

Furthermore, hRSV infection causes an inhibition of IFN α/β production by plasmacytoid DCs [51], which is thought to be due to the ability of both NS1 and NS2 proteins to impair the activation of the type I IFN response cascade. NS1 protein inhibits the phosphorylation of Interferon Regulatory Factor 3 (IRF-3), thereby inhibiting the activation of the IFN gene promotor and blocking the JAK/STAT signaling pathway. On the other hand, the NS2 protein interacts with RIG-I preventing the activation of IRF-3 [62,63] (Figure 1). Likewise, DCs infected with NS1- and NS2-deficient hRSV displayed higher expression of cell surface DC maturation markers, such as CD80, CD83, CD86 and CD38, as compared with DCs challenged with wild type hRSV. Herein, it is thought that NS1 and NS2 inhibit DC maturation by interfering with the activation of the type I IFN response. This notion is supported by the observation that DC maturation induced by NS1- and NS2-deficient hRSV is suppressed by IFN neutralization [64]. Consistently, hRSV induces only low to moderate maturation in human DCs, which is observed both in infected DCs as well in neighboring cells [65]. Further, although hRSV can promote maturation in mouse DCs, these cells are rendered incapable of activating naive T cells [66,67], which is consistent with an inefficient T cell response against hRSV infection [68]. It has been suggested that inefficient T cell activation by hRSV-infected DCs results from an impaired assembly of the immunological synapse between DCs and T cells [69]. Interestingly, the N protein of hRSV has been shown to be involved in the inhibition of immunological synapse assembly (Figure 2) [70]. Therefore, both inhibition of DC maturation and an inefficient T cell response during hRSV infection could explain the reduced IFN-γ production observed in bronchiolitis patients.

Both CD4^+^ and CD8^+^ T cells have been shown to be crucial for the establishment of an efficient anti-hRSV immunity [71]. Interestingly, these immune cells play a pivotal role during hRSV infection and can be either beneficial or detrimental for the host. Along these lines, human cytotoxic CD8^+^ T cells (CTLs) have been shown to recognize peptides derived from N, SH, F, M and NS1 proteins bound to MHC class I molecules and thereby contribute to the clearance of the virus [72,73]. However, it has also been suggested that depletion of CD8^+^ T cells can reduce the hRSV-associated severity in mice [74]. In addition to effector CD4^+^ and CD8^+^ T cells, regulatory T (Treg) can contribute to modulating hRSV infection. This notion is supported by the observation that depletion of CD4^+^FOXP3^+^CD25^+^ cells leads to an exacerbated inflammation pathology with higher weight loss and neutrophil infiltration into the lungs upon hRSV infection [75,76].

## 3. Prophylaxis against hRSV Infection

Children hospitalized because of severe cases of hRSV-associated pneumonia are a major public health burden worldwide. Prophylaxis against hRSV infection is intended to prevent hRSV infection-derived illness during virus outbreaks or after a potential exposure to the virus.

### 3.1. Antibody-Mediated Prophylaxis/Passive Immunization

#### 3.1.1. Current Monoclonal Antibody against hRSV

Currently, palivizumab (Synagys^®^, Medimmune, Inc., Gaithersburg, MD, USA), an IgG_1_ humanized monoclonal antibody, is the only licensed therapy to prevent severe lung disease caused by hRSV in high-risk infants. High-risk factors for hRSV disease include prematurity, bronchopulmonary dysplasia, congenital health disease, cancer, cystic fibrosis, down syndrome, neuromuscular syndrome and immune deficiency syndromes [77]. Palivizumab neutralizes an epitope of the hRSV F protein and has been demonstrated to inhibit the cell-to-cell fusion and the transcription of the virus [78]. Up to a 55% reduction in hospitalizations for high-risk infants during hRSV outbreaks can be obtained by the use of palivizumab [79,80]. Despite proven effectiveness, palivizumab requires intramuscular monthly injections throughout the hRSV outbreak, which is costly and unaffordable for most public health systems (near US$ 780 for the 50 mg vial and US$ 1416 for 100 mg, with a 15 mg/kg recommended dosage). In addition to the high cost, adverse events including hypersensitivity reactions have been reported in infants treated with palivizumab [81].

#### 3.1.2. Other Monoclonal Antibodies against hRSV

To improve safety and cost/efficacy, different approaches have been explored to improve the efficiency and specificity of the passive prophylaxis against hRSV (Table 1). One promising result was motavizumab, also known as MEDI-524, a monoclonal antibody derived from palivizumab by modifying the complementary determining regions. This monoclonal antibody showed increased affinity to the F protein as compared to palivizumab [82]. Motavizumab displayed a higher neutralizing activity and an improved capacity to prevent hRSV pathology compared to palivizumab in animal models [83]. Moreover, clinical trials to evaluate safety and pharmacokinetics of motavizumab obtained results equivalent to palivizumab [84,85,86]. However, no significant reduction of viral loads nor severity of hRSV-caused illness were found in infected infants treated with motavizumab, as compared with placebo controls [87]. It is important to mention that skin rashes, as an adverse event in motavizumab treated patients, were observed in all studies [84,85,88]. In 2010, the Food and Drug Administration (FDA) declined to license motavizumab to treat high-risk hRSV infants, in part because this monoclonal antibody offered the same efficacy as palivizumab. Due to these previous results with monoclonal antibodies, currently, molecular strategies are focused on improving the neutralizing capacity but also on reducing the observed adverse effects. Promising results with derivatives of motavizumab have been reported [89,90]. These monoclonal antibodies contain a modified Fc region, are well tolerated and show up to 100 days of half-life in human subjects [89]. Such an extended half-life together with higher levels of hRSV neutralizing antibodies as compared to palivizumab has also been reported with an anti-hRSV prefusion F monoclonal antibody, known as Medi8897 [90]. A phase II clinical trial in healthy preterm infants is currently in progress [91]. Due to the fact that both F and G are on the virus surface, the G protein has also been explored as an antigen to target hRSV. Indeed, anti-hRSV G monoclonal antibodies were also able to reduce pulmonary inflammation associated with hRSV in BALB/c mice [92,93]. However, to date, no clinical results have been reported for antibodies that target this protein as an hRSV antigen.

#### 3.1.3. Maternal Immunization against hRSV

Another strategy for vaccine research against hRSV has been maternal immunization. It was previously demonstrated that RSV-neutralizing antibodies are transferred efficiently through the placenta from pregnant women to newborns [94]. Consistently, studies in cotton rats showed that pups gestated by hRSV-primed mothers were protected from viral replication in the lungs [95]. Further, maternal immune protection was also observed in lambs after immunization with a subunit vaccine based on the F protein of hRSV with an adjuvant (ΔF/TriAdj). Promisingly, those newborns that received maternal antibodies showed less virus production and lung pathology as compared with control animals [96]. Immunization with a vaccine has already been found to be safe and immunogenic in healthy women of childbearing age in a phase II clinical trial [97]. This recombinant F nanoparticle formulation is currently on phase III clinical trial on women of childbearing age during their third-trimester of pregnancy and a phase III clinical trial in older adults with this formulation has been recently completed [98,99].

### 3.2. Active Immunization

The first approach to prevent hRSV was the formaldehyde-inactivated hRSV vaccine, which caused an exacerbated respiratory disease characterized by excessive inflammation with a Th-2 biased response [100]. Indeed, up to 80% of hospitalizations in children receiving this vaccine were reported [101,102]. Therefore, after that negative experience, therapies and vaccines approaches against the hRSV have been focused on achieving high immunogenicity but without promoting the exacerbated respiratory disease. Approximately sixty hRSV vaccine candidates are undergoing preclinical or clinical development at the moment [103]. A summary of the vaccines currently under research to prevent the hRSV infections is shown in Table 1.

#### 3.2.1. Live Attenuated Vaccines

The strategy of live attenuated vaccines was explored early to prevent hRSV disease. Current live attenuated vaccines have been engineered by reverse genetics. For instance, the deletion of M2-2 protein was used to obtain three candidates of attenuated vaccines [133]. One of them, known as hRSV LID ΔM2-2, has already shown stability, hRSV attenuation and anti-F serum IgG antibody responses in phase I clinical trials [109]. The other two candidates with the deletion of the M2-2 protein are currently under evaluation in phase I clinical trials [103]. Further, attenuating NS2 gene deletion has been explored as a strategy to develop a live attenuated RSV vaccine [110], which is currently in phase I clinical evaluation. Likewise, codon-reoptimization of NS1, NS2 and G, together with the deletion of SH proteins led to the generation of a stable attenuated hRSV vaccine, known as OE4 [134], which showed protection and lack of disease enhancement in mice and cotton rats [134].

#### 3.2.2. Recombinant Vector Vaccines against hRSV

In addition to live attenuated vaccines, recombinant vectored vaccines have also been explored for generating hRSV vaccines. For instance, Parainfluenza virus type 3 (PIV3) has been used as a vector to express hRSV proteins of interest [135,136]. Thus, recombinant bovine/human PIV3 (rB/HPIV3) expressing the G and F proteins can induce high anti-hRSV serum titers as well as protection against the virus in hamsters [136,137]. Recently, a partially stabilized prefusion form of the F protein was included in the genome of rB/HPIV3 to achieve higher quality of anti-hRSV-neutralizing antibodies in hamsters and rhesus monkeys than the previous formulation [106]. Also, the PIV type 5 (PIV5) has been shown to be an effective vector to express F and G proteins of hRSV, herein offering protection against the virus in mice [132].

Another successful recombinant approach to generate recombinant vaccines against hRSV was the Bacille Calmette-Guérin (BCG), which can efficiently express hRSV proteins [71,107]. BCG is an attenuated strain of *Mycobacterium bovis* and by itself displays a strong immunogenic capacity that promotes the production of Th-1 type cytokines, which are required to reduce hRSV dissemination and disease. Based on these properties, BCG was engineered to express various hRSV antigens [71]. Importantly, immunization with recombinant BCG strains expressing M2 or N proteins conferred protection against hRSV in mice and led to an early T cell recruitment to the lungs. Immunized animals showed no lung damage, lower viral loads and a Th1/Th2 balance after a challenge with hRSV [71,107]. Furthermore, a single dose of rBCG-N-hRSV manufactured under Good Manufacturing Practices (GMP) was shown to maintain those immunological properties and promoted long-lasting immunity in mice [108].

Furthermore, adenovirus has also been used as a vector to express hRSV antigens. Up to seven hRSV vaccine candidates have used adenovirus as a vector [103]. Adenovirus vectors expressing the hRSV F protein were immunogenic in mice and in cotton rats [126,127,128]. Further, the vaccine known as PanAd3-RSV showed protection against hRSV in calves [138] and a Phase I clinical trial has been recently completed [131]. On the other hand, baculovirus expressing the F protein in combination with virus-induced signalling adaptor reduced Th-2 responses and the immunopathology associated to hRSV infection [139,140]. Moreover, the F protein of hRSV and the hemagglutinin-neuraminidase protein of PIV were inserted into the Sendai virus genome to generate a vaccine inducing long-lasting protection against both hRSV and PIV in cotton rats [141]. Finally, a modified vaccine Ankara virus expressing hRSV proteins was shown to protect against virus infection in macaques [129,130] and to be safe and immunogenic in adults. Phase III clinical trials are currently ongoing for this vaccine [142].

#### 3.2.3. Virus-Like Particles (VLPs) as Vaccines for hRSV

Virus-like particles (VLPs), which consist of viral proteins assembled without genetic material, have also been used as a vaccine strategy against hRSV [114,115]. Thus, it was shown that VLPs containing the matrix protein of the human metapneumovirus (hMPV) together with the recombinant postfusion and prefusion F hybrids of hRSV promoted high levels of neutralizing antibodies, a Th-1 mediated response and protected against lung RSV infection [114]. Likewise, a combination of VLPs containing F and G proteins together with F-encoding plasmid DNA was found to be protective against hRSV disease by reducing weight loss and lung inflammatory damage [143]. Moreover, the bacterium-like particle (BLP) technology has also been used to develop a mucosal vaccine by including the F protein as an antigen to protect against hRSV. Such a RSV BLP vaccine reduced virus titers and resulted in higher titers of F-specific IgG in sera from cotton rats and mice challenged with hRSV [116]. Further, a Phase I clinical trial is in progress to assess the safety, reactogenicity and tolerability of two intranasal dose levels of this vaccine [117]. Further, the SH protein has also been underscored recently as a new target for vaccination against hRSV. As a vaccine candidate approach, a peptide derived from the SH ectodomain (SHe) was conjugated to the keyhole limpet hemocyanin [144]. This vaccine showed protection against hRSV and induced high levels of SH-specific IgGs in cotton rats and mice, however, sera from these animals showed no neutralizing capacity against hRSV [144]. Further, a phase I clinical trial was conducted with an hRSV vaccine containing the SH antigen and a novel adjuvant DepoVax or SH antigen co-administered with aluminum hydroxide, showing safety in vaccinated subjects [145].

Nanomedicine has appeared in the last few years to contribute to hRSV research vaccines. The reason for using such a technology is because it has been postulated that the density of the immunogens could be key for triggering the humoral immune response [146]. Nanoparticles technology can assemble several antigens in sizes ranging from 2 to 200 nm, thereby creating a high density “cocktail antigen”. Specifically, a high density of viral antigens facilitates antibody secretion and the formation of memory B cells [146]. Along these lines, a nanoparticle vaccine based on the F protein of hRSV was shown to be well tolerated in a Phase I clinical trial. Further, this vaccine was immunogenic in humans and led to high microneutralization antibody titers against the hRSV A Tracy and hRSV B strains. Further, this trial showed that the F hRSV nanoparticle vaccine induced an immune response against the epitope antigen site II by adding palivizumab in competitive assays [147]. In addition, this vaccine formulation was also used for maternal immunization as previously described [97]. Other VLPs with nanoparticles expressing hRSV glycoproteins also showed immunogenicity and the authors of the study demonstrated that alveolar macrophages are responsible for preventing detrimental infiltration by neutrophils, eosinophils and T cells, as well as mucus and inflammatory cytokine production [148]. Soluble nanorings composed of N protomers containing the epitope of the hRSV F targeted by palivizumab, known as N-FsII-nanorings, are another strategy [120]. Mice immunized with N-FsII-nanorings were protected from hRSV-caused disease, however, they showed no detectable neutralizing antibodies [120]. Moreover, patches loaded with N-nanorings have been found to protect against hRSV in pigs and have been demonstrated to be delivered efficiently through the skin and to reach Langerhans cells [121].

## 4. Concluding Remarks

The severe cases of pneumonia associated to hRSV infection are still a major public health burden worldwide and currently there are no licensed vaccines available. The only licensed treatment to prevent hRSV infections is the Palimizumab, an anti-F hRSV protein humanized monoclonal antibody that can reduce hRSV-associated hospitalizations by up to 55%, however, at a high cost [149]. Nowadays, several studies are in progress to generate a vaccine against hRSV, including live attenuated, viral-like particles and passive immunizations. Nevertheless, currently, there are few studies on human clinical trials or that have proven long-lasting immunity. Thus, new vaccines and therapies are urgently needed to reduce the high rate of hospitalizations caused by hRSV.

## Figures and Tables

**Table 1:** Current research on therapies and vaccines against hRSV infection.

Name	Strategy	Preclinical/Clinical studies	References
Motavizumab/MEDI-524	Anti-F Monoclonal antibody	Better neutralizing activity than palivizumab. Phase I, II and III clinical studies show similar results to palivizumab and some adverse events.	[84,85,86,87,88]
Motavizumab/MEDI-8897	Anti-F Monoclonal antibody	High levels of hRSV neutralizing antibodies. Phase I clinical studies show good tolerance and extended half-life of the antibody. Phase II in healthy preterm infants is ongoing.	[89,90,91]
mAb 131-2G	Anti G Monoclonal antibody	Reduces pulmonary inflammation in BABL/c mice.	[92,93]
Recombinant F nanoparticle	Anti-F Polyclonal	Maternal immunization has been shown to protect lambs and cotton rat newborns. Immunogenic in healthy women of childbearing age (Phase I and II studies). Phase III in women in their third-trimester of pregnancy is ongoing and phase III is completed in older adults.	[95,96,97,98,99]
Combination of RSV F VLP, G VLP, and RSV F DNA	RSV F DNA prime and VLPs containing F and G boost	Induces hRSV F specific IgG2a antibodies, neutralizing antibodies and prevents lung disease in BALB/c mice.	[104]
SV pcD-F	DNA vaccine encoding RSV-F protein	Topical vaccine induces cellular and mucosal immune response and reduces cell infiltration to the lungs in BALB/c mice.	[105]
rB/HIPV3	Recombinant parainfluenza virus (rB/HPIV3) expressing the G and F RSV proteins	High quality hRSV-neutralizing antibodies in hamsters.	[106]
rBCG-N-hRSV	BCG expressing N RSV protein	Protection against hRSV infection in mice with reduction of inflammation, hRSV specific T cell response and RSV antibodies in serum.	[71,107,108]
RSV LID ΔM2-2	Live attenuated vaccine, M2-2 RSV protein deleted	Immunogenic in chimpanzees. Phase I clinical study showed hRSV attenuation and RSV F serum IgG antibody responses.	[109]
RSV D46 cpΔM2-2	Live attenuated vaccine, M2-2 RSV protein deleted	Phase I in progress.	[103]
RSV Medi ΔM2-2	Live attenuated vaccine, M2-2 RSV protein deleted	Phase I in progress.	[103]
RSV ΔNS2 Δ1313	Live attenuated vaccine, NS2-2 RSV protein deleted	Demonstrated a stable formulation. Phase I in progress.	[110]
RSV cps2	Attenuated cold-passaged respiratory syncytial virus	Phase I in progress.	[103]
SeVRSV	Sendai virus (SeV)-based live intranasal vaccine that expresses the full length RSV fusion (F) gene	Protects cotton rats from hRSV challenge in a cotton rat maternal antibody model.	[111]
Delta-G RSV	Recombinant RSV lacking the G gene	Induces long-lasting protection against hRSV challenge and resulted in no detectable replication of hRSV in lungs and nasal washes in cotton rats.	[112]
OE4	Live attenuated vaccine, codon-reoptimization of RSV NS1, NS2 and G and deletion of SH proteins	Proven stability and no enhanced disease in cotton rats and mice.	[100]
NE-RSV	Nanoemulsion-inactivated RSV	Prevents hRSV-immunopathology and promotes Th-1/Th-17 responses in BALB/c mice	[113]
VLPs F	VLPs containing F RSV protein	High levels of neutralizing antibodies, Th-1 mediated response and protects against lung hRSV infection	[114]
VLPs F and G	VLPs containing F and G proteins	Protects against RSV disease by reducing cell infiltration to the lung, weight loss and lung damage.	[115]
DPX-RSV-SH	VLPs containing SH protein	Protection in cotton rats and mice. Phase I clinical trial showed safety.	[116,117]
RSV BLP	Bacterium-like particles (BLP)s containing F antigen	Protection in cotton rats and mice.	[118,119]
N-FsII-nanorings	Nanorings able to display the epitope of the human RSV F antigenic site	Protects against hRSV disease, however, they did not find detectable neutralizing antibodies	[120]
Viaskin®-N	Patches loaded with N-nanorings	Protects against hRSV in pigs and is delivered efficiently through the skin and reaches Langerhans cells.	[121]
G+CSA	Recombinant G protein with cyclosporine A	Induces Treg cells, controlling the hRSV-immunopathology.	[116]
RSV F	RSV fusion protein stabilized in the native prefusion conformation	Induces neutralizing antibodies and prevents viral challenge in cotton rats.	[117]
RSV G	G protein polypeptide and peptide vaccination	Prevents hRSV pathology and inhibit hRSV replication in mice.	[122]
RSV F protein	RSV postfusion F protein	Protection against hRSV challenge and antibody responses in BALB/c mice.	[123]
RSV-PreF	RSV protein F vaccine engineered to maintain prefusion conformation.	hRSV neutralizing antibody responses in Phase I clinical trial.	[124]
DNA RSV	DNA expressing soluble hRSV-F in combination with an AdV expressing the same antigen	Systemic DNA prime-tonsillar booster immunization regimen and induces the recruitment of hRSV-F-specific T cells to and/or expansion of the T cells in the respiratory tract in non-human primates	[125]
Ad5.RSV-F	Adenovirus expressing F RSV protein	Immunogenic in mice and cotton rats. Phase I currently in progress.	[126,127,128]
MVA	Modified vaccine Ankara virus expressing hRSV proteins	Protection in macaques. Safe and immunogenic in adults. Phase III currently in progress.	[129,130,131]
rPIV5-RSV-F and rPIV5-RSV-G	Vaccine based on Parainfluenza virus 5 (PIV5)	Generation of serum neutralizing mice and no enhanced disease upon hRSV challenge in mice.	[132]
PanAd3-RSV	Vaccine based on Simian adenovirus	Phase I clinical trial in progress.	[131]
